# Contraception in the ED: Understanding Education and Opportunities for Clinicians to Advise Patients

**DOI:** 10.5811/westjem.48376

**Published:** 2026-04-08

**Authors:** Hannah B. Lewis, Teagan R. McCarthy, Dara Kass, Jennifer L. Kahoud

**Affiliations:** * Thomas Jefferson University, Sidney Kimmel Medical College, Philadelphia, Pennsylvania; † Saint Francis Hospital, Department of Emergency Medicine, Hartford, Connecticut; ‡ Thomas Jefferson University Hospital, Department of Emergency Medicine, Philadelphia, Pennsylvania

## Abstract

**Introduction:**

The United States faces a high rate of unwanted pregnancies. Despite this, many people continue to face barriers to accessing contraception. The emergency department (ED) can help bridge these gaps, but emergency clinicians must first feel comfortable offering contraceptive services. In this study, we sought to determine emergency clinician comfort in prescribing contraceptives and educating patients on their use. We also gauged clinician interest in receiving education specifically geared toward contraceptive care.

**Methods:**

We conducted an online survey of ED residents, attendings, and advanced practice clinicians at Thomas Jefferson Univerity Hospital and affiliates in both the urban and suburban setting. Questions focused on current practices and interest in an educational session on contraceptive care in the ED.

**Results:**

We received 106 responses representing clinicians from 12 hospitals (estimated response rate 20%). While 61% of respondents reported that they offered contraceptive services less than once a month, 64% reported they were comfortable educating patients on the topic and 51% were comfortable providing prescriptions. Of those comfortable prescribing, 84% stated they would be more comfortable after an educational session, while 58% of those currently not comfortable prescribing believed that education would help (*P* < .01). Perceived benefit of education was also dependent on age, with clinicians < 35 years of age more likely to perceive a benefit (*P* < .01), and job title, with residents more likely to perceive a benefit (*P* = .04).

**Conclusion:**

Our data suggest that many emergency clinicians are open to offering contraceptive services, but lack of education may serve as a barrier. Although limited by self-selection bias, this study demonstrates a robust interest in overcoming this barrier within our sample group. Future work will aim to implement clinician education and assess for translation to clinical practice with the goal of increasing access to contraceptive services.

## INTRODUCTION

Over 40% of all pregnancies in the United States are unintended.^1^–^3^ Only 5% of these pregnancies occur in women who correctly use contraceptives, indicating that improving access to and education about contraception may reduce rates of unintended pregnancy.^2^ Preventing unintended pregnancy reduces morbidity and mortality related to birth while improving women’s access to education, workforce participation, and overall economic stability.^4^,^5^ Additionally, interventions aimed at improving contraceptive access can reduce government spending resulting from unintended births, which has amounted to $12.5 billion in a single year.^5^ The overturning of Roe v Wade by the U.S. Supreme Court in 2022 has accelerated the need for such interventions as more individuals will be legally compelled to carry unintended pregnancies to term.

One approach to increasing access may be offering contraception in the emergency department (ED). There are 6,000 EDs in the U.S., which see approximately 130 million people per year.^6^ People of childbearing age may account for > 20% of these visits.^7^ With an “open-door” policy and 24-hour services, the ED has the potential to reach patients who may otherwise lack access to contraception and, therefore, be at higher risk for unintended pregnancy. Interventions in the ED have proven to be successful in response to other areas of unmet public health needs.^8^–^10^ Offering contraception in the ED could be an effective way to increase access and prevent unintended pregnancies.

Potential barriers to this approach include knowledge gaps among clinicians and current attitudes about scope of emergency medicine (EM) practice. Research on this topic in the field of EM is lacking, but studies in other non-gynecological specialties show opportunities to close knowledge gaps that may serve as a barrier to care.^11^,^12^ These studies, focused on residents in internal medicine and other primary care specialties, show contraceptive knowledge is both objectively and subjectively inadequate.^11^,^12^ Furthermore residents attribute their lack of providing contraceptive counseling in practice to these inadequacies.^11^ The potential need for emergency clinician education and receptiveness to such education must be addressed before any interventions can be implemented.

Our objective in this study was to determine emergency clinicians’ comfort levels in offering ED-based contraceptive care and to evaluate their interest in receiving further education on the topic. Results will inform future efforts to support contraceptive services in the ED.

## METHODS

### Study Design

This study consisted of a 14-question, online questionnaire (Qualtrics International Inc, Provo, UT) sent by electronic mail to listservs of emergency clinicians at Thomas Jefferson Univerity Hospital and its affiliates between June–August 2023. The emails lists were provided by the ED coordinators at each location. Approximately 500 questionnaires were sent out. No reminder emails were sent or incentives offered. It was approved by the Thomas Jefferson Univerity Hospital Institutional Review Board as an exempt protocol. Consent was obtained as part of the questionnaire before proceeding with the study questions.

### Study Population

Eligible participants were active clinicians at 12 clinical sites at or affiliated with Thomas Jefferson Univerity Hospital. The sites included three urban and nine suburban institutions consisting of both Level I and II trauma centers spanning the states of Pennsylvania, Delaware, and New Jersey. Clinicians included attending physicians, residents, and advanced practice practitioners (APP).

**Population Health Research Capsule**

What do we already know about this issue?

*Increased contraceptive access can alleviate morbidity and mortality associated with unintended pregnancy, but knowledge gaps prevent clinicians from offering these services*.

What was the research question?

*How comfortable are ED clinicians in prescribing contraceptives and is there interest in further education?*

What was the major finding of the study?

*Contraception is rarely offered in the ED; 70% of clinicians believe education would increase current prescription practices*.

How does this improve population health?

*By preventing unintended pregnancies, increased contraceptive access may reduce maternal morbidity and mortality and improve economic stability*.

### Survey Development and Measures

The survey was designed to capture knowledge, attitudes, and current practices with regard to contraception in the ED. Initial questions were created around these domains. The survey was developed iteratively with review by experts in EM, including review for comprehension and responder burden. Questions covered the following topics: current prescribing practices; comfort offering contraceptive services; perceived benefit of further education; and content and format preferences for education. Clinicians were also asked to complete basic demographic questions. The final survey is included as Supplement A.

Three questions addressed comfort in educating patients on birth control options, prescribing birth control, and calling consults for birth control prescriptions. Those who indicated they were comfortable prescribing birth control were asked to select which forms they felt comfortable prescribing (oral contraceptive pills, contraceptive patch, vaginal ring, contraceptive implant, intrauterine device [IUD], or injectable Depo-Provera). Clinicians were then asked to indicate whether they believed their comfort in educating or prescribing would change following an educational session.

To inform potential education session design, a secondary outcome of this study, the remaining questions asked survey respondents to indicate what contraceptive types they would want included, as well as to rank their preferences for session length and format. Clinicians interested in providing additional information to inform contraception efforts were given the option to provide their contact information. Otherwise, respondents remained anonymous. Because the topic of contraception can be personal, responses to all questions were optional. Not every survey respondent answered every question; however, we included the results of all submitted surveys. Because not all questions were answered by all respondents, some questions have a smaller number of total responses.

### Statistical Analysis

Descriptive analyses included response counts/frequencies, percentages, and means. We calculated all percentages based on the total number of survey respondents (n = 106). To assess for differential views in perceived benefit of an educational session, we stratified respondents based on reported age, sex, job title, and current prescription frequency. We used chi-square tests to determine whether these categories had an effect on responses. Statistical significance was defined by a *P* value < .05.

## RESULTS

A total of 106 clinicians responded to the survey. Based on our best estimate of the number of eligible respondents, this accounts for an approximate response rate of 20% as defined by American Association for Public Opinion Research Response Rate 2 formula.^19^ Of the respondents, 74 (70%) were predominantly White, 47 (44%) were between 25–34 years of age, and 58 (54%) were attending physicians. Fifty respondents identified as male, while 52 identified as female. Thomas Jefferson Univerity Hospital had the greatest number of responses (n = 45; 42%).

### Current Practices

While 55 (51%) clinicians reported they would feel comfortable prescribing birth control in the ED, the majority (n = 32, 30%) reported they did not offer contraceptive services in the ED or offered them less than once a month (n = 33, 31%). Of those who reported they were comfortable prescribing birth control, the majority were comfortable with oral contraceptive pills; however, their comfort level in prescribing decreased when asked about other contraceptive measures. When asked how they felt with reference to educating patients about contraception, 68 (64%) reported that they felt comfortable doing so.

### Future Comfort with Educational Session

When asked whether they would be more likely to educate patients about birth control if they received an educational session on best prescribing practices, 68 (64%) respondents agreed that they would. Of the clinicians who previously stated they were not comfortable educating women and girls, 20 (57%) stated they would be more comfortable after receiving an educational session. Of the clinicians who stated they were comfortable educating patients, 48 (71%) stated they would be more comfortable after receiving an educational session.

Similarly, 74 (70%) respondents believed they would be more likely to prescribe birth control if they received an educational session on contraceptives and best prescribing practices, and 28 respondents (58%) who reported not being comfortable prescribing contraception believed an educational session would enhance their comfort level. In comparison, of the respondents who stated that they were currently comfortable prescribing contraception, 46 (84%) reported that an educational session would help their comfort level. This difference was found to be statistically significant (*P* < .01) (Table). Chi-squared analyses of stratified data showed that clinicians ≤ 34 years of age were more likely to indicate that education would benefit them compared to their older colleagues (*P* < .01). Respondents were naturally bisected at 34 years of age, given that approximately half fell below this age. Additionally, residents and APPs were more likely than their attending counterparts to indicate increased comfort in educating patients following clinician education (*P* = .04).

### Type of Educational Session

Clinicians indicated they would prefer an educational session to be in person, take as little time as possible, and include information about oral contraceptive pills, contraceptive patch, vaginal ring, contraceptive implant, IUD, and the Depo shot.

## DISCUSSION

The ED has great potential to increase contraceptive access. Previous studies have shown that adults and adolescents of childbearing age receiving emergency care are receptive to the idea.^13^,^14^ Our data suggest that while emergency clinicians are open to offering contraception, they rarely do. Given knowledge gaps that exist within many non-obstetric specialties regarding contraceptive care, clinician discomfort may stem in part from a lack of education. This study supports this hypothesis and establishes a need for education among emergency clinicians.

Although our results showed that most respondents felt comfortable offering contraceptive services, this finding was more robust in regard to educating patients on options rather than actually prescribing contraception (64% vs 52%). Moreover, those who indicated comfort in prescribing were mainly referring to oral contraceptive pills as opposed to other methods. In addition, among those who reported being comfortable with educating patients, their comfort was limited to discussing oral conceptive pills, rather than other contraceptive options. This is in line with findings from similar surveys of non-gynecologic clinicians, which indicate that comfort prescribing contraception does not necessarily apply to methods such as IUD insertion.^12^,^15^ Oral contraceptive pills are less effective at preventing pregnancy than other methods and have side effects that may limit their use.^16^ Furthermore, reproductive autonomy is best supported by offering individuals the fullest range of options possible. Therefore, it is important for clinicians to feel confident offering alternatives to oral contraceptives. Taken together, these findings indicate a need for education that is prescription-focused and covers a wide range of methods.

The primary outcome of this study was clinicians’ self-perceived need for education. We found that 70% believe they would be more comfortable prescribing contraceptives after further education. Interestingly, most clinicians who believed they would benefit from education were those already comfortable prescribing contraceptives and offering contraceptive education. This tells us that although there is an interest in education, the clinicians who are most likely to participate in such education are already open to offering these services. Those who are not comfortable may be less likely to attend an educational session due to little perceived benefit.

Moreover, younger clinicians were more likely than those > 35 to indicate interest in education. Similarly, residents and APPs were more likely than attendings to indicate interest. This presents a substantial challenge given that younger physicians and residents are often practicing under the supervision of older attending counterparts. Contraceptive services are unlikely to be offered if supervising clinicians are not comfortable doing so. While disinterest in continuing education opportunities may arise due to lack of time or perceived clinical relevance, these factors are not necessarily specific to attending physicians.^17^ Therefore, to achieve meaningful change, efforts to address clinician knowledge must also better characterize and target underlying attitudes that may serve as a barrier.

This study also provided us with insight into preferred methods of education. We believe that using this information to inform future educational session design may increase use. Future work will focus on developing and implementing clinician education and assessing for changes in clinical practice and impact on patient outcomes within our patient population, as was similarly done by Liang and colleagues.^18^

## LIMITATIONS

Although the survey was reviewed by emergency clinicians actively involved in women’s health research, survey development was limited by lack of input from experts in the field of contraceptive care. Furthermore, our survey was limited by reliance on outdated demographic descriptors including the use of “Caucasian” instead of “White” and “gender” in place of “sex.” We also recognize that use of the terms “women” and “girls” in our survey does not accurately reflect all patients with the ability to become pregnant. All survey questions are also subject to reader interpretation despite efforts to remain objective.

Additionally, there are limitations to the data itself and what we could extrapolate from it. We collected no data on what clinicians perceive as current barriers to prescribing contraception, the average number of patients of childbearing age seen by them, or specific comfort in initiating a conversation on contraception. These topics would all be useful to address in follow-up studies. Moreover, we did not further stratify job title data based on age to assess whether the difference between respondents < 35 and > 35 years of age was truly an age-based difference vs a role-based difference as most clinicians < 35 are APPs/residents. That being said, most of our respondents identified as young attendings, yet attendings were still less likely to perceive education as beneficial, from which we infer that being an attending and age are independent factors affecting perceived benefit of contraceptive education.

This study was also influenced by self-selection bias because it was based on a voluntary survey sent to emergency clinicians via email. Clinicians who filled out the survey may have been more interested in the idea of contraceptive education than those who did not. It is also possible that younger clinicians were more likely to fill out the survey given that our participants were mostly < 45 years of age. This survey was also limited to Thomas Jefferson Univerity Hospital and affiliates; therefore, responses may not be generalizable to other EDs. Additionally, as survey respondents were contacted through listserv, we do not have an updated list of the total number of clinicians who were contacted. Because of this, we do not know our precise response rate, which is a significant limitation in the generalizability of our study.

## CONCLUSION

Our study suggests that many ED clinicians within the Thomas Jefferson Univerity Hospital system are interested in contraceptive education. Creating an educational model according to their expressed preferences (short, in-person session) could help expand contraceptive access, which is paramount in the wake of the 2022 Dobbs Supreme Court decision. Further research should be done to explore the effects of such education.

## Supplementary Information





## Figures and Tables

**Table:** Clinician interest in contraceptive education, based on their demographic profile, when asked the following questions:

Would you be more likely to prescribe birth control if you had an educational session on contraceptives and best prescribing practices?			
Gender* (n = 102)	Yes (%)	No (%)	*P* = .75
Male (n = 50)	37 (74)	13 (26)	
Female (n = 52)	37 (71)	15 (29)	
Job Title (n = 101)			*P* = .12
Attending physician (n = 58)	37 (64)	21 (36)	
Resident (n = 32)	27 (84)	5 (16)	
Other (advanced practice practitioner) (n = 11)	8 (73)	3 (27)	
Age (years) (n = 103)			*P* = .02
0–34 (n = 47)	39 (83)	8 (17)	
≥ 35 (n = 56)	35 (62.5)	21 (37.5)	
Current contraceptive prescribing practices (n = 99)			*P* = .22
Once a month or more than once a month (n = 34)	27 (79)	7 (21)	
Less than once a month (n = 65)	44 (68)	21 (32)	
Currently comfortable prescribing birth control in the ED (n = 103)			*P* < .01
Yes (n = 55)	46 (84)	9 (16)	
No (n = 48)	28 (58)	20 (42)	
Would you be more likely to educate women and girls on birth control options if you had an educational session on contraceptives and their uses?			
Gender* (n = 102)	Yes (%)	No (%)	*P* =.89
Male (n = 50)	33 (66)	17 (34)	
Female (n = 52)	35 (67)	17 (33)	
Job Title (n = 101)			*P* = .04
Attending physician (n = 58)	32 (55)	26 (45)	
Resident (n = 32)	25 (78)	7 (22)	
Other (advanced practice practitioner) (n = 11)	9 (82)	2 (18)	
Age (years) (n = 103)			*P* < .01
0–34 (n = 47)	39 (83)	8 (17)	
≥ 35 (n = 56)	29 (52)	27 (48)	
Currently comfortable educating women in the ED (n = 103)			*P* = .17
Yes (n = 68)	48 (71)	20 (29)	
No (n = 35)	20 (57)	15 (43)	

* Our survey is limited by the use of outdated demographic descriptors including the use of “gender” in place of “sex.”

*ED*, emergency department.
