# Supplementary material for: Contraception in the ED: Understanding Education and Opportunities for Clinicians to Advise Patients
Source: West J Emerg Med. 2026 Apr 8;27(3):725–30. doi: 10.5811/westjem.48376 (PMC13246203; doi:10.5811/westjem.48376)
Supplement: Supplementary file 1 [file wjem-27-725-s001.docx]

**Supplement A. Questionnaire**

1. What gender do you identify as?
   1. Male
   2. Female
   3. Other
   4. Prefer not to say
2. What is your age?
   1. 0-24
   2. 25-34
   3. 35-44
   4. 45-54
   5. 55-64
   6. 65+
3. Please specify your ethnicity
   1. Caucasian
   2. African-American
   3. Latino or Hispanic
   4. Asian
   5. Native American
   6. Native Hawaiian or Pacific Islander
   7. Two or more
   8. Other/Unknown
   9. Prefer not to say
4. What is your job title?
   1. Intern resident
   2. Second year resident
   3. Third year resident
   4. Attending Physician
   5. Advanced Practice Providers
   6. Other
5. Which emergency medicine department do you work at? *(Multiselect)*
   1. Jefferson center city
   2. Jefferson Methodist Hospital
   3. Einstein Philadelphia
   4. Einstein Montgomery
   5. Jefferson Abington
   6. Jefferson Abington Lansdale
   7. Jefferson Bucks
   8. Jefferson Cherry Hills
   9. Jefferson Frankford
   10. Jefferson Stratford
   11. Jefferson Torresdale
   12. Jefferson Washington Township
   13. Nemours
   14. Other
6. How often do you provide contraceptive services to patients in the ED (prescriptions, consults, referrals, etc.)
   1. Every day
   2. Multiple times a week
   3. Once a week
   4. 2-3 times per month
   5. Once a month
   6. Less than once a month
   7. Never
   8. Other
7. Would you feel comfortable educating women and girls about birth control options in the ED?
   1. Yes
   2. No
8. Would you feel comfortable prescribing birth control in the ED?
   1. Yes
   2. No
9. *(If yes to 8)* What types of birth control would you feel comfortable prescribing in the ED? ​​*(Multiselect)*
   1. Oral Contraceptive Pills
   2. Contraceptive Patch
   3. Vaginal Ring
   4. Contraceptive Implant
   5. Intrauterine Device
   6. DMPA (Depo shot)
10. Would you feel comfortable calling a consult to prescribe birth control?
    1. Yes
    2. No
11. Would you be more likely to educate women and girls on birth control options if you had an educational session on contraceptives and their uses?
    1. Yes
    2. No
12. Would you be more likely to prescribe birth control if you had an educational session on contraceptives and best prescribing practices?
    1. Yes
    2. No

*Questions 13-15 only apply if survey participant answered yes to question 11 and/or 12.*

1. What types of birth control methods would you like to be included in this session? *(Multiselect)*
   1. Oral Contraceptive Pills
   2. Contraceptive Patch
   3. Vaginal Ring
   4. Contraceptive Implant
   5. Intrauterine Device
   6. DMPA (Depo shot)
2. Please rank how long you would like this educational session to be. (*Rank answer choices 1-7)*
   1. As short as possible
   2. 30 Minutes
   3. 1 Hour
   4. 3-5 Hours
   5. 1 Day
   6. Multiple Days
   7. Other
3. Please rank your preferred type of educational session. *(Rank answer choices 1-5)*
   1. In person lecture
   2. Synchronous zoom
   3. Asynchronous zoom
   4. Pre-recorded module
   5. Other
4. Are you interested in learning more about this program and becoming a project champion?
   1. Yes
   2. No
5. *(If yes to 16)* Please include your full name and email.
