# Supplementary material for: Contraception in the ED: Understanding Education and Opportunities for Clinicians to Advise Patients
Source: West J Emerg Med. 2026 Apr 8;27(3):725–30. doi: 10.5811/westjem.48376 (PMC13246203; doi:10.5811/westjem.48376)
Supplement: Supplementary file 2 [file wjem-27-725-s002.docx]

**Supplement B. Demographics Data**

| **Total Respondents** | 106 |
| --- | --- |
| **Ethnicity** |  |
| Caucasian | 74 |
| African-American | 2 |
| Latino or Hispanic | 5 |
| Asian | 13 |
| Two or more | 4 |
| Other/Unknown/Prefer not to say | 5 |
| **Gender** |  |
| Male | 50 |
| Female | 52 |
| Prefer Not to Say | 1 |
| **Age (Years)** |  |
| 25-34 | 47 |
| 35-44 | 34 |
| 45-54 | 11 |
| 55-64 | 8 |
| 65+ | 3 |
| **Job Title** |  |
| Intern Resident | 7 |
| Second Year Resident | 13 |
| Third Year Resident | 10 |
| Attending Physician | 58 |
| Advanced Practice Providers | 11 |
| Other | 3 |
